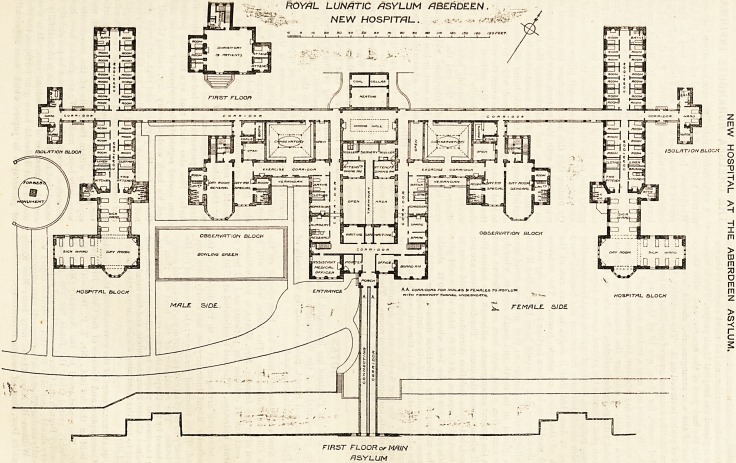# Hospital Construction

**Published:** 1899-08-12

**Authors:** 


### HOSPITAL CONSTRUCTION.

### NEW HOSPITAL AT THE ABERDEEN ASYLUM.

Many of the District and Royal Asylums of Scotland have adjuncts or annexes which are called hospitals, and are used for the sick and acute cases. Medical superintendents across the Border seem to be convinced of the usefulness of such hospitals, but it can hardly be said that their English brethren hold the same views; and for ourselves we think that a properly-designed public asylum should be entirely self-contained, unless we make exception of a detached block for infectious diseases, and, possibly, a second one for private patients. We give the ground plan of the most recently built of these hospitals, namely, that attached to the Royal Asylum, Aberdeen. In this instance the hospital is about 120 feet from the main building, is attached to it by a twin corridor, and, hence, is free from one of the objections (the chief one) which would strike the critic when studying the question. Here, also, the corridor appears to have been cleverly designed. It has a tramway tunnel underneath—and it must form a good boundary line between the gardens used by the male patients and those used by the women.

The hospital has a southern or rather a south-western aspect. The administration block is in the centre, and it contains medical officers' rooms, "research" room, surgery, board-room, dining-room, sculleries, attendants' rooms, bath-rooms, and various other offices; but no kitchen is shown, from the absence of which we imagine that the cooking is done in the main building, an arrangement quite free from objection provided all the accessories are favourable, as they seem to be here.

East and west of the central block are the men's and women's observation wards. They are connected to the centre by "exercise" corridors, which have on their south sides verandahs and on their north sides conservatories. This is a very good idea, as in wet or cold weather what to do with the patients is often an insoluble problem. The conservatories, from their northern position, may not get much sun, although they will always be useful as lounges, and they would have looked better if the partitions between them and the exercise corridors had been of glass.

The day-room for special cases is narrow and shut in on two sides, but this drawback is lessened by a large window in front and another at the back which looks into an enclosed court. The general day-room is of better shape and has a fine bay window, but it is practically shut in on three sides, hence, cross ventilation must be difficult to carry out. Both of these day-rooms have their w.c.'s contiguous, and there are no ventilating passages between them and the wards. Indeed, this arrangement has been carried out throughout the hospital, and we need hardly say that we look upon it as entirely wrong. A ventilating passage is in all cases

NEW HOSPITAL AT THE ABERDEEN ASYLUM.

essential. Northwards of the observation block runs the connecting corridor, and at right angles to the corridor is placed the hospital block proper. This is incomparably the best part of the annexe, and, with the exception of the w.c. arrangements, should have a fair meed of praise bestowed on it. The architects have not fallen into the common error of providing too few single-bedded rooms, for there are 18 of these to each sick block. We have not seen a section nor a roof plan, so that we cannot say whether the corridor has sufficient lantern lights, or whether the single room doors are provided with movable fanlights. So, also, we have not a block plan showing the ground space at disposal; but if space permitted, we are of opinion that the connecting corridor should not have intersected the hospital ward where it does, but that it ought to have run at the back of the block of single rooms. The isolation block would then have been further from the day-rooms and dormitories, and it would have been better if this dormitory had been larger, so that windows could have been placed in its north and south aspects, whereby cross-ventilation would have been more efficient than with the present arrangement, although we notice the small windows near the doors. The dimensions are not figured, but these isolation dormitories would seem to be about 16 ft. square. At 12 ft. high this would give a little over 1,500 ft. per bed, whereas 2,000 ft. should be the minimum for infectious disease, for which we presume these isolation blocks are intended.

The administration-block is three storeys high, and is occupied by the staff. The observation blocks are of two storeys; the first floors contain dormitories, rooms for attendants, and single-bedded rooms. The latter are placed over the ground floor w.c.'s, an arrangement which may be free from danger, but which is not quite pleasant.

## Figures and Tables

**Figure f1:**